# Toward a versatile toolbox for cucurbit[*n*]uril‐based supramolecular hydrogel networks through *in situ* polymerization

**DOI:** 10.1002/pola.28667

**Published:** 2017-06-22

**Authors:** Ji Liu, Cindy Soo Yun Tan, Yang Lan, Oren A. Scherman

**Affiliations:** ^1^ Melville Laboratory for Polymer Synthesis Department of Chemistry, University of Cambridge Cambridge CB2 1EW United Kingdom; ^2^ Faculty of Applied Sciences Universiti Teknologi MARA Kota Samarahan Sarawak 94300 Malaysia

**Keywords:** cucurbit[n]uril, host‐guest systems, hydrogels, *in situ* polymerization, self healing, supramolecular hydrogel network, toughness

## Abstract

The success of exploiting cucurbit[*n*]uril (CB[*n*])‐based molecular recognition in self‐assembled systems has sparked a tremendous interest in polymer and materials chemistry. In this study, polymerization in the presence of host‐guest complexes is applied as a modular synthetic approach toward a diverse set of CB[8]‐based supramolecular hydrogels with desirable properties, such as mechanical strength, toughness, energy dissipation, self‐healing, and shear‐thinning. A range of vinyl monomers, including acrylamide‐, acrylate‐, and imidazolium‐based hydrophilic monomers, could be easily incorporated as the polymer backbones, leading to a library of CB[8] hydrogel networks. This versatile strategy explores new horizons for the construction of supramolecular hydrogel networks and materials with emergent properties in wearable and self‐healable electronic devices, sensors, and structural biomaterials. © 2017 The Authors. Journal of Polymer Science Part A: Polymer Chemistry Published by Wiley Periodicals, Inc. J. Polym. Sci., Part A: Polym. Chem. **2017**, *55*, 3105–3109

## INTRODUCTION

The ability to control hierarchical assembly of macromolecular units into high‐fidelity structures and high performance systems across multiple length scales provides access to a wide variety of artificial adaptive materials.^1^, ^2^, ^3^, ^4^, ^5^, ^6^, ^7^, ^8^, ^9^, ^10^, ^11^, ^12^, ^13^ Recently, supramolecular building components, such as cucurbit[*n*]uril (CB[*n*])‐mediated host‐gust complexes, have drawn enormous attention in the context of self‐assembled materials and systems.^5^, ^14^ Such dynamic yet strong supramolecular interactions have been used as a reversible linking motif to bring together two separate entities, leading to the formation of homo/heteroternary complexes in an aqueous environment. Subsequently, stimuli‐responsiveness or other functions can be realized in such dynamic systems. To date, this strategy has been further applied in the construction of numerous assemblies, such as supramolecular hydrogel networks.^14^ Many previously reported CB[*n*] hydrogel networks have exploited the mixtures of two complementary‐functionalized polymers in aqueous conditions. Upon the addition of CB[8] host molecules, hydrogel networks displaying moderate viscoelastic properties are readily constructed through the dynamic CB[8] host‐guest complexation.^5^, ^15^, ^16^ However, this strategy faces several challenges, such as the limited aqueous solubility of CB[8], exceptionally viscous solutions of high molecular weight polymer, heavy reliance on synthetic or functionalized natural resources (such as cellulose), moderate mechanical properties, as well as limited potential for scale‐up and manipulation.

**Figure 1:**
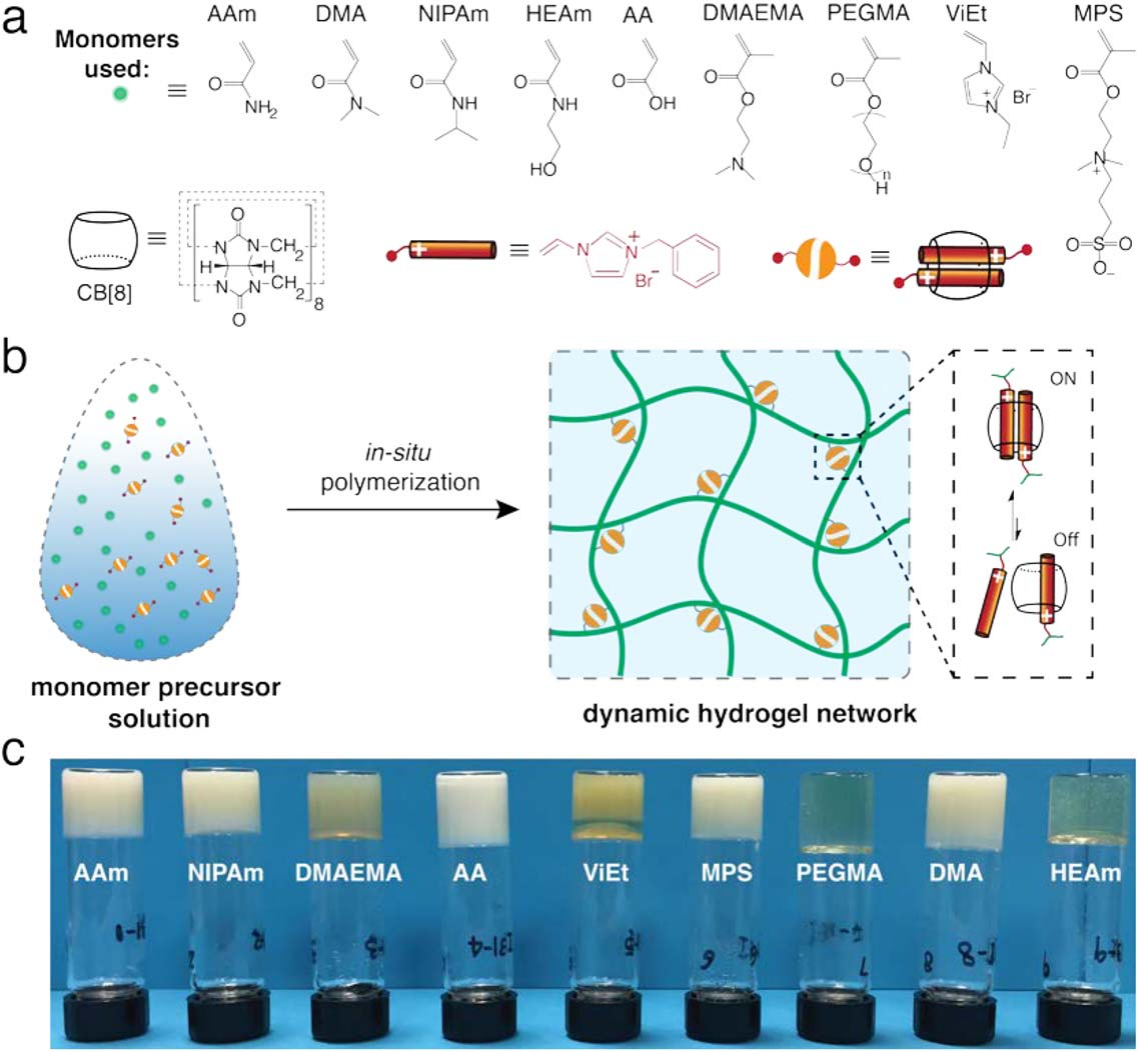
(a,b) *In situ* polymerization of supramolecular polymer networks in the presence of macrocyclic host cucurbit[8]uril (CB[8]) and polymerizable guest molecules (1‐benzyl‐3‐vinylimidazolium bromide) and a hydrophilic co‐monomer. The CB[8] supramolecular ternary conjugates serve as dynamic crosslinkers, leading to a network structure after photo‐initiated polymerization at room temperature. They also act as sacrificial bonds that rupture (OFF state) under deformation and dissipate energy, which can further re‐form (ON state), resulting in the self‐healing of a macroscopic supramolecular network. (c) Photographs of the supramolecular hydrogel networks from different co‐monomers, as well as the corresponding inverted‐vial demonstration of their viscosities. Note: acrylamide (AAm), *N*‐isopropylacrylamide (NIPAm), 2‐(dimethylamino)ethyl methacrylate contains (DMAEMA), acrylic acid (AA), 1‐vinyl‐3‐ethylimidazolium bromide (ViEt), 3‐[2‐(methacryloyloxy)ethyl](dimethyl)ammonio‐1‐propanesulfonate (MPS), poly(ethylene glycol) methacrylate (PEGMA), dimethylacrylamide (DMA), and *N*‐hydroxyethyl acrylamide (HEAm). [Color figure can be viewed at wileyonlinelibrary.com]

In recent progress toward the facile construction of CB[8] hydrogel networks with desirable mechanical properties,^14^ we recently reported the photo‐initiated *in situ* polymerization of CB[8] supramolecular hydrogel networks from a monomer precursor solution (Fig. 1).^17^, ^18^ A polymerizable guest (1‐benzyl‐3‐vinylimidazolium bromide), serving as a *supramolecular crosslinker* upon complexation with CB[8] in a 2:1 manner [Fig. 1(b)], was incorporated to build the network structures. The dynamic CB[8] host‐guest interactions contribute to the networks' toughness, as well as energy dissipation through reversible association/dissociation of the complexes. Compared with previously reported CB[*n*] hydrogels,^15^, ^16^ such an *in situ* polymerization approach allows for a substantially higher loading of CB[8] in the systems, as well as overall monomer concentration, giving access to a higher degree of non‐covalent crosslinking and mass fraction of the resultant hydrogel networks, which further results in superior mechanical performances. In this report, a range of vinyl monomers, including acrylamide‐ (AAm), acrylate‐, and imidazolium‐based hydrophilic monomers [Fig. 1(a)], are incorporated as the polymer backbones to obtain a library of CB[8] hydrogel networks.

## EXPERIMENTAL

### Materials

Monomers like AAm, dimethylacrylamide (DMA), *N‐*isopropylacrylamide (NIPAm), *N*‐hydroxyethyl acrylamide (HEAm), acrylic acid (AA), 2‐(dimethylamino)‐ethyl methacrylate (DMAEMA), poly(ethylene glycol) methacrylate (PEGMA), 1‐vinyl‐3‐ethyl‐imidazolium bromide (ViEt), and 3‐[2‐(methacryloyloxy)ethyl](dimethyl)ammonio‐1‐propanesulfonate (MPS) are purchased from Sigma‐Aldrich. Unless specified, all the chemicals used in this work were used without further purification.

### Instrumentation

^1^H NMR (500 MHz) spectra were collected on a Bruker Avance QNP 500 MHz ultrashield spectrometer, equipped with a 5‐mm BBO ATM probe with a z‐gradient. Photo‐irradiation was performed on a photo‐reactor with UV lamps (365 nm, power density of 4.8 mW cm^−2^). Rheological tests were performed using a Discovery Hybrid Rheometer (DHR)‐2, a controlled stress hybrid rheometer from TA Instruments fitted with a water bath, which was set to various temperatures. The disc‐shaped samples with thicknesses of 0.5 mm and diameter of 20 mm were used and surrounded by a vendor supplied solvent trap to mitigate solvent (water) loss during the measurements, and results were analyzed using TA Instruments TRIOS software. Dynamic oscillatory strain amplitude sweeps were conducted at a frequency of 10 rad s^−1^. Dynamic oscillatory frequency sweep measurements were conducted at a 1% strain amplitude, between 0.01 and 100 rad s^−1^, while temperature frequency sweep tests were carried out with a shear strain of 1% and shear frequency of 60 rad s^−1^, on a ramp at an interval of 5 °C between 0 and 80 °C.

### Synthesis of 1‐Benzyl‐3‐Vinylimidazolium Bromide

Benzyl bromide (50 mmol) was added drop wise into a solution of vinyl imidazole (50 mmol) in diethyl ether (100 mL) at 0 °C within 10 min, and left for another 16 h at room temperature. The crude product was filtered off, giving a gray powder that was washed with diethyl ether, and then dried under vacuum until a constant weight, reaching to a yield of almost 100%.

^1^H NMR (500 MHz, D_2_O, 298 K, *δ*, ppm): 10.68 (s, 1H, —N^+^—C**H**—N—), 7.17–7.91 (m, 7H, aromatics), 7.22 (dd, 1H, —N—C**H**=CH_2_), 5.89 (dd, 1H, —N—CH=CH—**H**_trans_), 5.52 (s, 2H, Ph—C**H**_2_—N^+^—), 5.16 (dd, 1H, —N—CH=CH—**H**_*cis*_).

### Synthesis of CB[8]‐Based Supramolecular Hydrogel Networks

Typically, for the AAm‐based hydrogel network, predetermined amounts of 1‐benzyl‐3‐vinylimidazolium (5 mol %)), CB[8] (2.5 mol %), and AAm (95 mol %) were dissolved in 2 mL Milli‐Q H_2_O (18 mΩ). After purging with nitrogen for 30 min, 0.03 mol % of photo‐initiator was added to the monomer solution. The monomer solution was exposed to UV irradiation (4.8 mW cm^−2^) for 6 h. The other monomer‐based hydrogel networks were prepared following the same strategy. The as‐obtained samples were used for measurement directly.

## RESULTS AND DISCUSSION

This work exploits the applicability of such *in situ* polymerization as a modular toolbox for CB[8] hydrogel networks with a broader scope of starting monomers. In addition to the AAm‐based CB[8] hydrogel network, we prepared a series of hydrogel networks with a variety of hydrophilic monomers following a similar protocol (Fig. 1). Three classes of vinyl monomers were used including (1) AAm‐based monomers including AAm, DMA, HEAm, and NIPAm, (2) acrylate‐based monomers such as AA, MPS, PEGMA, and DMAEMA, as well as (3) imidazolium‐based monomers such as ViEt. Photo‐initiated polymerization of each monomer precursor solution can readily generate supramolecular hydrogel networks as demonstrated with inverted‐vial tests [Fig. 1(c)]. In contrast, control polymerizations in the presence of CB[7] or in the absence of CB[*n*] result in high‐viscosity fluids, confirming the critical role of CB[8] host‐guest interactions in formation of the hydrogel networks.

**Figure 2:**
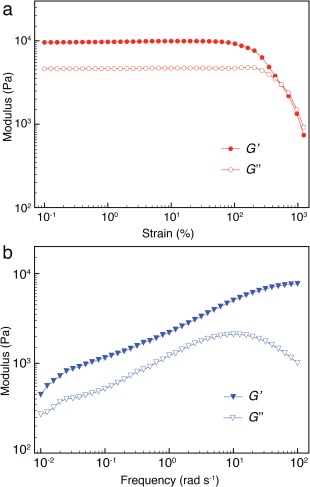
*G*′ and *G*″ values of the HEAm‐based CB[8] hydrogel network via dynamic room‐temperature (a) amplitude sweep (from 10^−1^ to 10^3^ % strain, 10 rad s^−1^) and (b) frequency sweep (from 10^−2^ to 10^2^ rad s^−1^, 1% strain). [Color figure can be viewed at wileyonlinelibrary.com]

To probe the network dynamics of these supramolecular hydrogel networks as well as quantify their mechanical properties, rheological characterization was performed. A representative strain‐dependent oscillatory rheology [Fig. 2(a)] of the HEAm‐based hydrogel network (solid fraction of 12 wt %, CB[8] crosslinking of 2.5 mol %)) displays an extremely broad linear viscoelastic region before structural failure at high strain (about 550%), indicating a wide processing regime and shear thinning behavior. Compared with previous CB[8] hydrogels with intermediate mechanical properties (i.e., with a plateau modulus of 10^1^–10^3^ Pa),^15^, ^16^ hydrogel networks with *G*′ > 10^4^ Pa can be easily obtained by tuning the overall solid fraction and/or dynamic crosslinking degree.

The frequency‐dependence of *G*′ and *G*″ confirms the elastic behavior of the supramolecular networks, as *G*′ is dominant across the whole range of frequencies applied [10^−2^–10^2^ rad s^−1^, Fig. 2(b)]. In those previous studies, the critical relaxation time (*β*) was estimated when *G*′ and *G*″ crossed over in an oscillatory frequency sweep, below which the hydrogel networks are elastic (*ω* > *β*), but turned to viscous fluids as the viscous component dominated (*ω* < *β*).^5^, ^15^, ^16^ Although *β* of the CB[8] hydrogel networks here could not be detected within the experimental timescales (10^−2^–10^2^ rad s^−1^), the principle of time‐temperature superposition (TTS) can extend the *β* value over a wider angular frequency range (10^−3^–10^3^ rad s^−1^).^15^, ^16^ On account of the larger number of dynamic crosslinks, higher solid mass fraction (> 10 wt %) and polymer chain entanglement, much slower relaxation after oscillatory perturbation (i.e., higher elasticity) was obtained for these CB[8] hydrogel networks, as evidenced by the higher moduli with *G*′ > *G*″ throughout the entire range of angular frequencies. Oscillatory dynamic rheological sweeps of all other hydrogel networks exploiting different monomers mentioned above are presented in the Supporting Information Figure S1–S8. On account of different monomer compositions, direct comparison between all the hydrogel networks was not carried out. A more detailed investigation on some specific systems may be presented in due course.

The temperature‐dependent rheological behavior was recorded up to 80 °C [Fig. 2(a)], above which evaporation of water from the CB[8] hydrogel networks in the parallel plate geometry may become problematic. Throughout the whole temperature range, *G*′ was stable before 40 °C, after which the network sample started to lose its mechanical properties. A decrease in the mechanical properties with increasing temperature can be intuitively attributed to the concomitant increase in the dissociation dynamics of the CB[8] host‐guest complexes at elevated temperature. This observation further proves that the CB[8] ternary complexes are responsible for the 3D networks and their corresponding viscoelastic properties. In the subsequent cooling process, the network samples gradually recovered their initial mechanical strength, which was not observed in previous hydrogel systems,^16^ indicating a high thermostability and reversibility.

**Figure 3:**
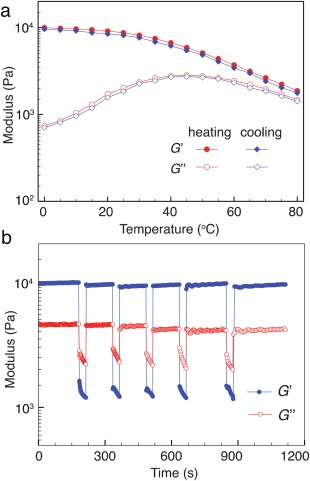
(a) Thermal stability of the HEAm‐based CB[8] hydrogel network during cyclic heating and cooling process between 0 and 80 °C; (b) continuous step‐strain measurements of the network sample at 20 °C (high‐amplitude oscillatory parameters: strain *γ* = 500%, angular frequency *ω* = 10 rad s^−1^, low‐amplitude oscillatory parameters: *γ* = 0.5%, *ω* = 10 rad s^−1^). [Color figure can be viewed at wileyonlinelibrary.com]

The capability of natural systems to heal cracks always involves an energy dissipative mechanism via sacrificial bonds, which can dynamically break and reform before fracturing the entire transient scaffold.^7^, ^8^, ^9^, ^10^, ^11^ Incorporation of dynamic interactions, as sacrificial bonds, can effectively increase the overall viscoelastic dissipation, but also impart the capability of recovering their initial strength after failure.^8^, ^11^, ^14^ Microscopic self‐healing behavior of a HEAm‐based hydrogel network was investigated by rheological step‐strain measurements, where mesoscale ruptures were induced at high strain, a critical parameter for processing and injection of soft materials. As shown in Figure 3(b), a high strain oscillatory sweep (*γ* = 500%) results in a quasi‐liquid structure (*G*″/*G*′ ∼ 2.0), where the hydrogel sample readily flows. However, when the network was subjected to a subsequent low‐magnitude strain sweep (*γ* = 0.5%), *G*′ immediately recovers its initial value at a quasi‐solid state (tan*δ* ∼ 0.5) in a few seconds following the stress‐induced flow. Interestingly, the rate and extent of recovery were nearly similar over several cycles of simultaneous breaking and reforming, highlighting the reversible and robust essence of such dynamically crosslinked networks. Therefore, reversible dissociation/association of the host‐guest complexes can effectively retard network failure, and ensure an astounding toughness.^17^, ^18^ Similar macroscopic self‐healing of the hydrogel network was also observed in tensile tests of AAm‐based CB[8] hydrogel networks,^18^ where complete recovery of the mechanical strength was reported after 5 h healing of two cut pieces at room temperature without the addition of any extra materials (Supporting Information Fig. S9).

As reported, such CB[8] hydrogel networks exhibited a toughness up to 2000 J m^−2^ (from Rivlin‐Thomas pure shear tests),^17^, ^18^ which is comparable to that of cartilage (1000 J m^−2^). As such, these tough hydrogel networks exhibit great potential as structural biomaterials for regenerative tissue engineering, similar to double networks reported by Gong and coworkers^19^ and graphene‐based hydrogels by Li and coworkers,^20^ to cite only a few. Moreover, this synthetic strategy allows for the flexible preparation of hydrogel networks over a wide range of mechanical strength, due to the applicability to the monomer precursor solutions with a range of monomer concentrations, which are not limited by solution viscosity. For example, hydrogel networks with intermediate strength (1–1000 Pa) can be readily obtained by tuning the overall solid fraction and crosslinking degree;^18^ such tuning gives rise to their potential use as moldable and injectable hydrogels for *in vivo* drug delivery platforms, similar to the polymer/particle hydrogel reported by Langer and coworkers.^21^

In addition to applications inspired by these networks' remarkable mechanical performance, mobile ions within the selected hydrogel networks enable the percolation of conductive pathways, thus extending their potential as highly stretchable ionic conductors and pressure sensors.^18^ Inherent ionic conductivity of these flexible yet tough transparent materials opens the possibility to construct robust interfaces between ionic/biological and ionic/electronic systems, similar to the aliginate‐based networks demonstrated by Suo and coworkers as well as Zhao and coworkers.^22^, ^23^ Finally, incorporation of positively‐charged monomers such as ViEt, negatively‐charged monomers such as AA, zwitterionic monomers (e.g., MPC), and other pH‐sensitive monomers (e.g., DMAEMA and AA) or thermosensitive monomers (e.g., NIPAm, DMAEMA, and PEGMA) can also enrich these CB[8]‐based supramolecular hydrogel networks with additional characteristics and emergent properties, which will be exploited in the near future.

## CONCLUSIONS

Since the first report on cucurbit[*n*]uril‐based hydrogels, dynamic CB[8] host‐guest complexation has been extensively exploited as modular building components, promoting its versatility in the preparation of specialty assemblies. In this work, the synthesis of CB[8] hydrogel networks via facile *in situ* polymerization of monomer precursor solutions, in the presence of preformed CB[8] host‐guest complexes, was demonstrated. This strategy readily generates a library of CB[8] hydrogel networks from diverse monomers such as acrylates, AAms, electrolytes, and zwitterions. It dramatically expands the scope of CB[*n*]‐based dynamic materials, becoming a primary choice for the preparation of functional supramolecular materials.

## Supporting information

Supporting Information

## References

[1] P. Egan , R. Sinko , P. R. LeDuc , S. Keten , Nat. Commun. 2015, 6, 7418. 26145480 10.1038/ncomms8418

[2] A. M. Kushner , Z. Guan , Angew. Chem. Int. Ed. 2011, 50, 9026–9057. 10.1002/anie.201006496 21898722

[3] J. R. Capadona , K. Shanmuganathan , D. J. Tyler , S. J. Rowan , C. Weder , Science 2008, 319, 1370–1374. 18323449 10.1126/science.1153307

[4] K. Liu , Y. Kang , Z. Wang , X. Zhang , Adv. Mater. 2013, 25, 5530–5548. 24038309 10.1002/adma201302015

[5] J. Liu , Y. Lan , Z. Y. Yu , C. S. Y. Tan , R. M. Parker , C. Abell , O. A. Scherman , Acc. Chem. Res. 2017, 50, 208–217. 28075551 10.1021/acs.accounts.6b00429 PMC5474693

[6] A. Miserez , T. Schneberk , C. Sun , F. W. Zok , J. H. Waite , Science 2008, 319, 1816–1819. 18369144 10.1126/science.1154117 PMC2754134

[7] P. Cordier , F. Tournilhac , C. Soulié‐Ziakovic , L. Leibler , Nature 2008, 451, 977–980. 18288191 10.1038/nature06669

[8] G. M. van Gemert , J. W. Peeters , S. H. Söntjens , H. M. Janssen , A. W. Bosman , Macromol. Chem. Phys. 2012, 213, 234–242.

[9] M. Nakahata , Y. Takashima , H. Yamaguchi , A. Harada , Nat. Commun. 2011, 2, 511. 22027591 10.1038/ncomms1521 PMC3207205

[10] Y. Chen , A. M. Kushner , G. A. Williams , Z. Guan , Nat. Chem. 2012, 4, 467–472. 22614381 10.1038/nchem.1314

[11] T. F. de Greef , E. Meijer , Nature 2008, 453, 171–173. 18464733 10.1038/453171a

[12] M. Zhang , D. Xu , X. Yan , J. Chen , S. Dong , B. Zheng , F. Huang , Angew. Chem. Int. Ed. 2012, 124, 7117–7121. 10.1002/anie.201203063 22653895

[13] X. Ji , Y. Yao , J. Li , X. Yan , F. Huang , J. Am. Chem. Soc. 2012, 135, 74–77. 23259828 10.1021/ja3108559

[14] J. Liu , C. S. Y. Tan , Y. Lan , O. A. Scherman , Macromol. Chem. Phys. 2016, 217, 319–332.

[15] E. A. Appel , F. Biedermann , U. Rauwald , S. T. Jones , J. M. Zayed , O. A. Scherman , J. Am. Chem. Soc. 2010, 132, 14251–14260. 20845973 10.1021/ja106362w

[16] C. S. Y. Tan , J. del Barrio , J. Liu , O. A. Scherman , Polym. Chem. 2015, 6, 7652–7657.

[17] J. Liu , C. S. Y. Tan , Z. Y. Yu , Y. Lan , C. Abell , O. A. Scherman , Adv. Mater. 2017, 29, 1604951. 10.1002/adma.201604951 28092128

[18] J. Liu , C. S. Y. Tan , Z. Y. Yu , N. Li , C. Abell , O. A. Scherman , Adv. Mater. 2017 DOI:10.1002/adma.201605325. 10.1002/adma.201605325 28370560

[19] T. Nonoyama , S. Wada , R. Kiyama , N. Kitamura , M. Mredha , T. Islam , X. Zhang , T. Kurokawa , T. Nakajima , Y. Takagi , K. Yasuda , J. P. Gong , Adv. Mater. 2016, 28, 6740–6745. 27184968 10.1002/adma.201601030

[20] J. Lu , C. Cheng , Y. S. He , C. Lyu , Y. Wang , J. Yu , L. Qiu , D. Zou , D. Li , Adv. Mater. 2016, 28, 4025–4031. 27031209 10.1002/adma.201505375

[21] E. A. Appel , M. W. Tibbitt , M. J. Webber , B. A. Mattix , O. Veiseh , R. Langer , Nat. Commun. 2015, 6, 6295. 25695516 10.1038/ncomms7295 PMC4651845

[22] C. Keplinger , J. Y. Sun , C. C. Foo , P. Rothemund , G. M. Whitesides , Z. Suo , Science 2013, 341, 984–987. 23990555 10.1126/science.1240228

[23] H. Yuk , T. Zhang , S. Lin , G. A. Parada , X. Zhao , Nat. Mater. 2016, 15, 190–196. 26552058 10.1038/nmat4463 PMC4762474

