# Supplementary material for: Toward a versatile toolbox for cucurbit[n]uril‐based supramolecular hydrogel networks through in situ polymerization
Source: J Polym Sci A Polym Chem. 2017 Jun 22;55(18):3105–9. doi: 10.1002/pola.28667 (PMC5575522; doi:10.1002/pola.28667)
Supplement: Supplementary file 1 — Supporting Information [file POLA-55-3105-s001.pdf]

Supporting Information:
Towards a Versatile Toolbox for Cucurbit[n]-based Supramolecular Hydrogel Networks Through in situ Polymerization

Ji Liu, [a] Cindy Soo Yun Tan, [a,b] Yang Lan [a] and Oren A. Scherman [a*]

E-mail: oas23@cam.ac.uk

[a] Melville Laboratory for Polymer Synthesis, Department of Chemistry, University of Cambridge, Lensfield Road, Cambridge CB2 1EW, UK.

[b] Faculty of Applied Sciences, Universiti Teknologi MARA, 94300 Kota Samarahan, Sarawak, Malaysia.

S.III. Supporting Results.

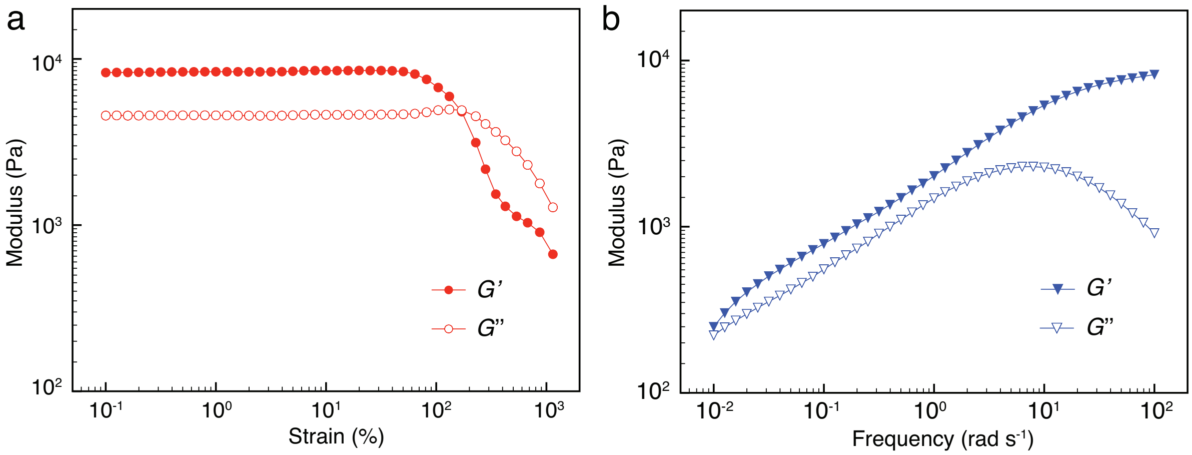

Figure S1: G' and G'' values of the AAm-based CB[8] hydrogel network *via* dynamic room-temperature amplitude sweep (**a**. from 10^{-1} to 10^{3} % strain, 10 rad s^{-1}) and frequency sweep (**b**. from 10^{-2} to 10^{2} rad s^{-1}, 1% strain).

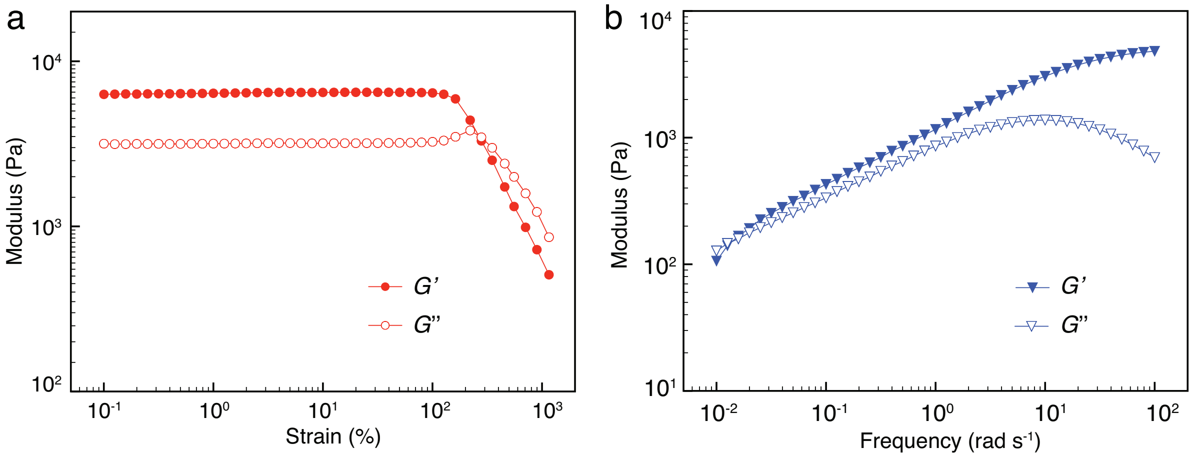

Figure S2: G' and G'' values of the *N*-isopropylacrylamide (NIPAm)-based CB[8] hydrogel network *via* dynamic room-temperature amplitude sweep (**a**. from 10^{-1} to 10^{3} % strain, 10 rad s^{-1}) and frequency sweep (**b**. from 10^{-2} to 10^{2} rad s^{-1}, 1% strain).

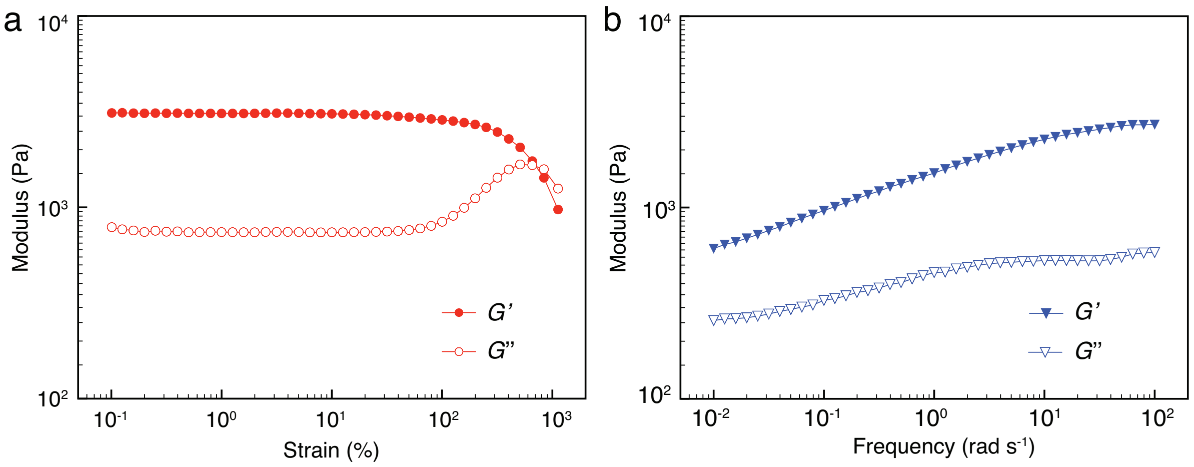

Figure S3: G' and G'' values of the 2-(dimethylamino)ethyl methacrylate-based CB[8] hydrogel network *via* dynamic room-temperature amplitude sweep (**a**. from 10^{-1} to 10^{3} % strain, 10 rad s^{-1}) and frequency sweep (**b**. from 10^{-2} to 10^{2} rad s^{-1}, 1% strain).

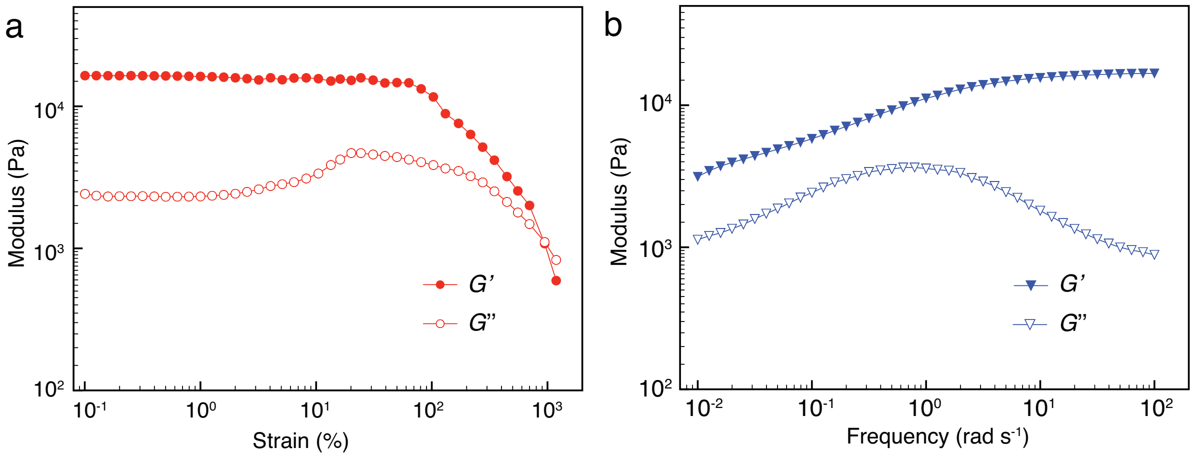

Figure S4: G' and G'' values of the acrylic acid (AA)-based CB[8] hydrogel network *via* dynamic room-temperature amplitude sweep (**a**. from 10^{-1} to 10^{3} % strain, 10 rad s^{-1}) and frequency sweep (**b**. from 10^{-2} to 10^{2} rad s^{-1}, 1% strain).

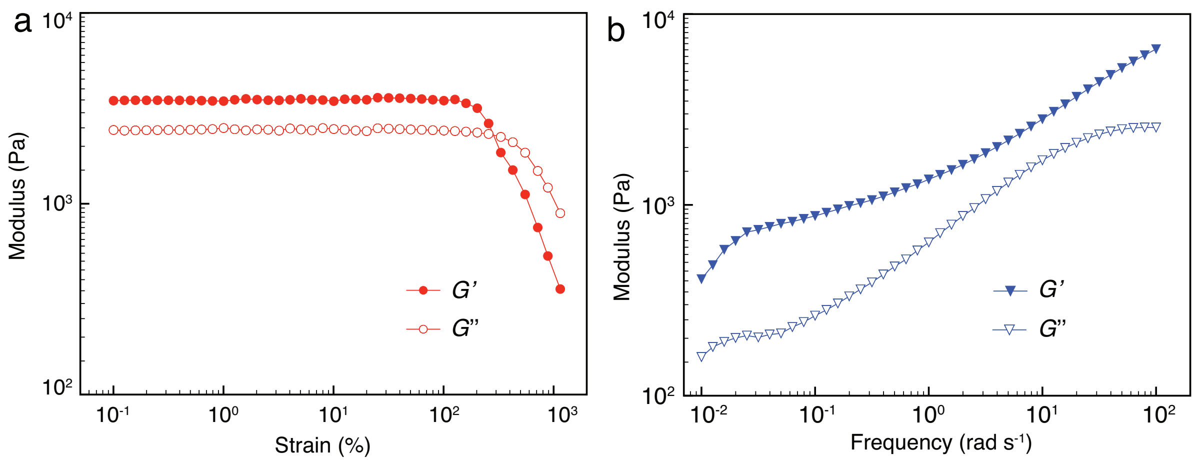

Figure S5: G' and G'' values of the 1-vinyl-3-ethylimidazolium bromide (ViEt)-based CB[8] hydrogel network *via* dynamic room-temperature amplitude sweep (**a**. from 10^{-1} to 10^{3} % strain, 10 rad s^{-1}) and frequency sweep (**b**. from 10^{-2} to 10^{2} rad s^{-1}, 1% strain).

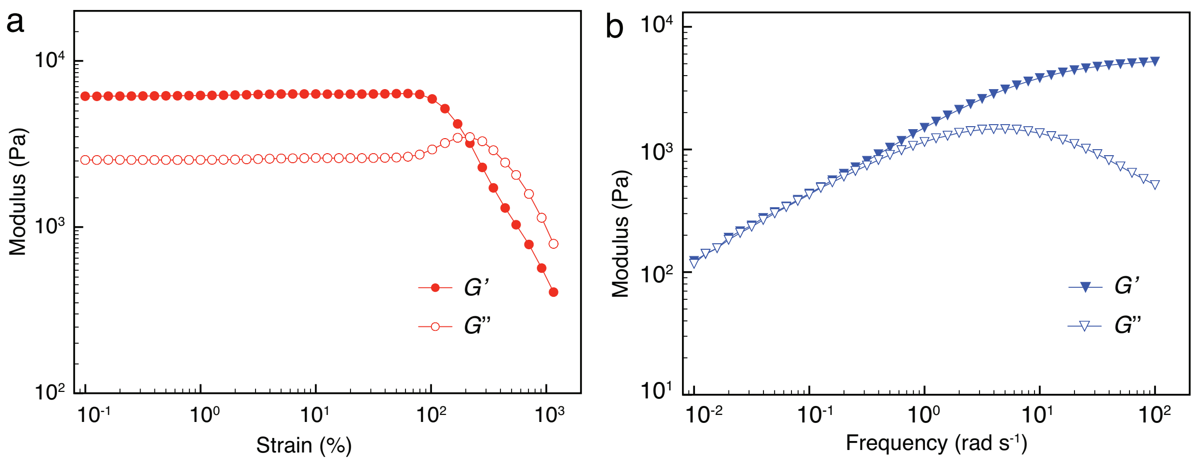

Figure S6: G' and G'' values of the 3-[2-(methacryloyloxy)ethyl](dimethyl)ammonio-1-propanesulfonate (MPS)-based CB[8] hydrogel network *via* dynamic room-temperature amplitude sweep (**a**. from 10^{-1} to 10^{3} % strain, 10 rad s^{-1}) and frequency sweep (**b**. from 10^{-2} to 10^{2} rad s^{-1}, 1% strain).

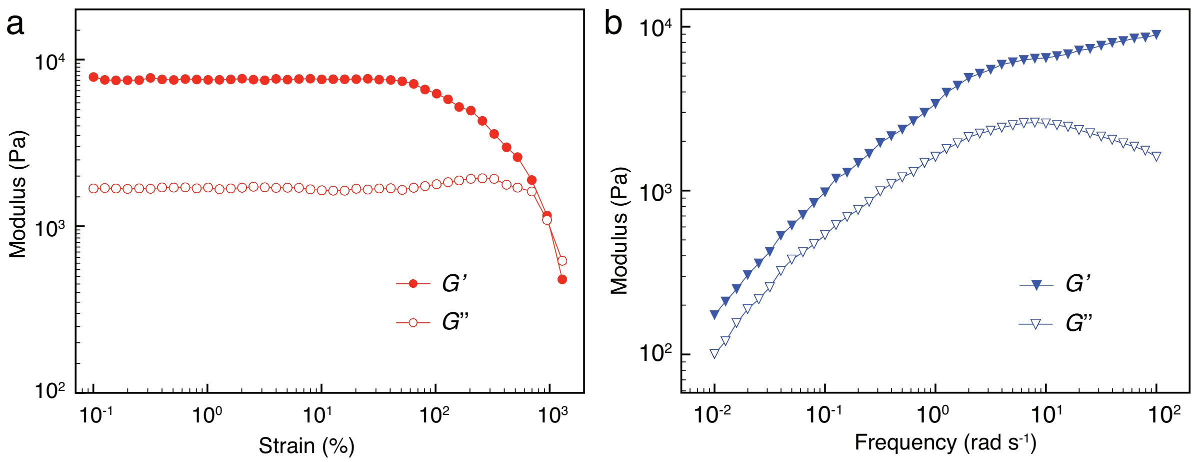

Figure S7: G' and G'' values of the poly(ethylene glycol) methacrylate (PEGMA)-based CB[8] hydrogel network *via* dynamic room-temperature amplitude sweep (**a**. from 10^{-1} to 10^3 % strain, 10 rad s^{-1}) and frequency sweep (**b**. from 10^{-2} to 10^2 rad s^{-1}, 1% strain).

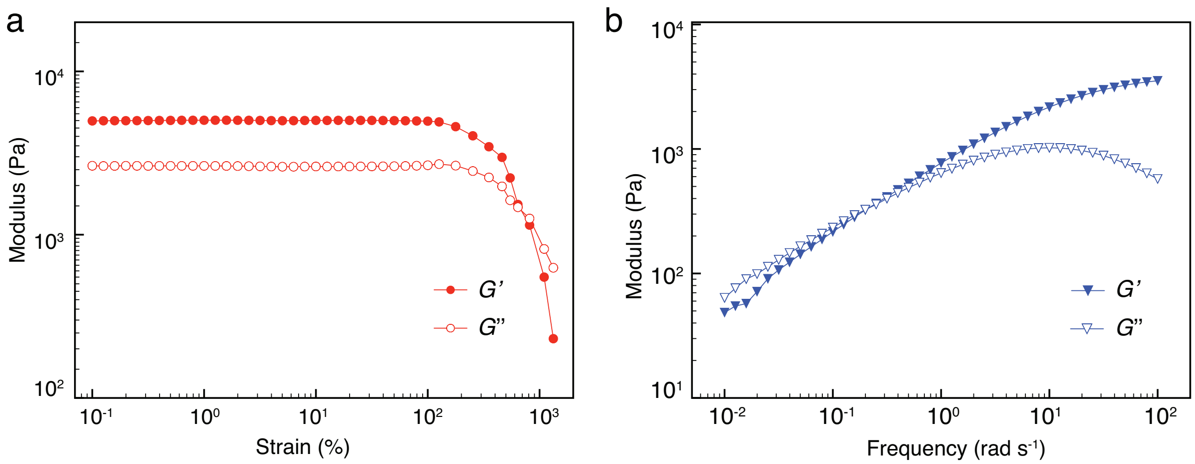

Figure S8: G' and G'' values of the dimethylacrylamide (DMA)-based CB[8] hydrogel network *via* dynamic room-temperature amplitude sweep (**a**. from 10^{-1} to 10^3 % strain, 10 rad s^{-1}) and frequency sweep (**b**. from 10^{-2} to 10^2 rad s^{-1}, 1% strain).

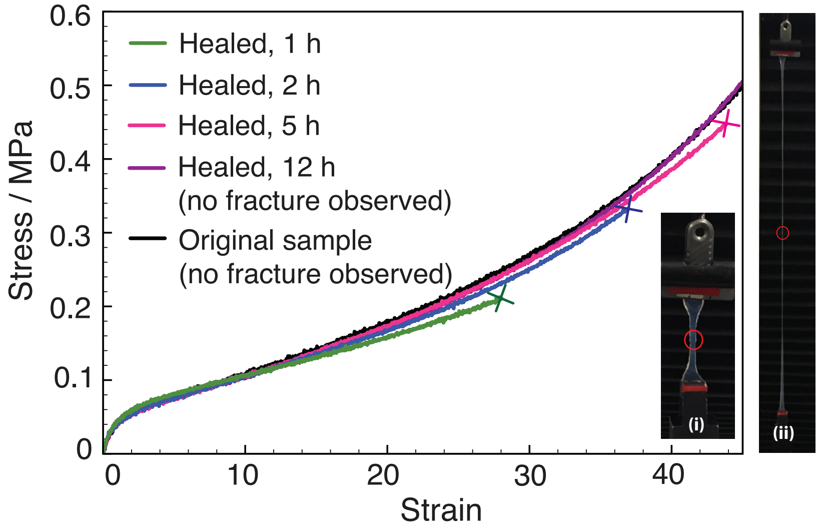

Figure S9: Stress-strain curves of the virgin and self-healed acrylamide-based CB[8] hydrogel network after different healing times at 25 °C (note: the original sample and self-healed sample after 12 h did not fracture at strain of 45×, which is the maximum strain achieved by the tensile machine); inset: photographs demonstrating the self-healed sample during stretching at a deformation ratio of $\lambda = 0$ (**i**) and $\lambda = 28$ (**ii**). The red circles refer to the cut location of a self-healed sample (1 h) before and after stretching to a strain of 28×. Adapted from Ref. 18 (O. A. Scherman, *et al.*, *Adv. Mater.* **2017**, in press, DOI:10.1002/adma.201605325).
